# Hemostatic efficacy of a novel Ca/P/S-based bone graft material and its preliminary clinical evidence in sternum closure procedures

**DOI:** 10.3389/fbioe.2026.1845027

**Published:** 2026-06-15

**Authors:** Jun-Neng Roan, Pao-Yen Lin, Jiin-Huey Chern Lin, Chen-Hsu Wang, Chun-Yu Liu, Chien-Ping Ju, Tai-Hua Yang

**Affiliations:** 1 Division of Cardiovascular Surgery, Department of Surgery, National Cheng-Kung University Hospital, College of Medicine, National Cheng-Kung University, Tainan, Taiwan; 2 Center for Biomaterials Research, National Cheng-Kung University, Tainan, Taiwan; 3 Department of Materials Science and Engineering, National Cheng-Kung University, Tainan, Taiwan; 4 Joy Medical Devices Corp., Kaohsiung, Taiwan; 5 Department of Biomedical Engineering, National Cheng Kung University, Tainan, Taiwan; 6 Department of Orthopedic Surgery, National Cheng Kung University Hospital, College of Medicine, National Cheng Kung University, Tainan, Taiwan

**Keywords:** bone regeneration, Ca-based bone substitute, fibrin fiber, hemostasis, inflammation

## Abstract

Median sternotomy is the standard approach for cardiac surgery. Still, it is often complicated by excessive marrow bleeding and impaired healing, particularly in frail patients, and conventional hemostatic agents can control bleeding but frequently compromise bone regeneration. We evaluated Ezechbone® Granule CBS-400, a fully synthetic Ca/P/S-based bone graft substitute, against commercially available calcium-based products to address this challenge. *In vitro* blood clotting assays showed that CBS-400 consistently achieved the lowest blood clotting index, indicating rapid clot formation. *In vivo* rat tibial implantation revealed denser and more stable fibrin fiber networks and markedly reduced white blood cell infiltration, highlighting superior clot stability and early biocompatibility compared with controls. Clinical pilot applications in high-risk sternotomy patients confirmed its translational relevance: CBS-400 reduced chest tube drainage, shortened drainage duration, and decreased intensive care unit stays while supporting stable sternal healing. These findings demonstrate that CBS-400 combines hemostatic efficacy with osteoregenerative potential, positioning it as a multifunctional biomaterial that may improve outcomes in complex surgical settings. Larger randomized studies are warranted to validate these results and define optimal clinical use.

## Highlights

- CBS-400 accelerates whole-blood clot formation *in vitro*.
- CBS-400 promotes dense, stable fibrin networks *in vivo* within 30 min.
- CBS-400 reduces acute leukocyte infiltration at early implantation.
- Pilot sternotomy cases suggest reduced drainage without impaired healing.

## 1 Introduction

Median sternotomy remains the gold standard for cardiac surgical access, with over one million coronary artery bypass grafting (CABG) procedures performed annually worldwide (Vervoort et al., 2023). Despite its technical maturity, postoperative management-particularly chest tube drainage-continues to play a pivotal role in determining outcomes. According to Wynne et al., average chest drainage in CABG patients reaches 1,300.6 ± 763.8 mL over 45.2 h, with a temporal decrease from 31 mL/h in the initial 8 h to 21 mL/h between 24 and 48 h postoperatively (Wynne et al., 2007). However, patients with frail sternal bone marrow present a unique clinical challenge. Their marrow cavities are highly susceptible to copious bleeding during sternotomy, which interferes with bone union and significantly increases the risk of mediastinitis-an infection with a reported mortality rate ranging from 14% to 47% (Sjogren et al., 2006). Christensen’s prospective cohort study further identified increased chest tube drainage as an independent predictor of elevated 30-day mortality (OR = 1.12 per 100 mL increase, p < 0.01) and other postoperative complications (Christensen et al., 2012).

In this high-risk population, the dual challenge of marrow bleeding and sternal instability necessitates special attention. Excessive bleeding not only fosters a nutrient-rich environment that triples mediastinitis incidence (Sjogren et al., 2006), but sternal malunion also disrupts normal respiratory mechanics, often requiring prolonged ventilator support. Indeed, over 81% of such patients remain intubated beyond 48 h, increasing susceptibility to ventilator-associated pneumonia and raising overall healthcare costs (Mueller et al., 2000). Conventional hemostatic agents, such as beeswax-based blends, alkylene oxide copolymers, and various soft tissue hemostats including thrombin, collagen, platelet-rich plasma (PRP), and fibrin sealants, have been employed to manage intraoperative bleeding. However, most of these agents focus solely on hemostasis without promoting bone healing, and adverse effects; for example, bone wax impairs osteogenesis and promotes chronic inflammation, gelatin and collagen may delay healing and raise infection risk, and bovine thrombin can elicit immunologic responses (Angelini et al., 1987; Nelson et al., 1990; Wellisz et al., 2008; Wellisz, 2012; Schmitz and Sodian, 2015; Tham et al., 2018; Mac et al., 2025).

Therefore, achieving both hemostasis and osteogenesis in a single biomaterial has become a priority. Existing calcium-based materials such as tricalcium phosphate (TCP)/hydroxyapatite (HA) composites (Lamas and Centella, 2013) and HA/polylactic acid (PLA) hybrids (Tham et al., 2018) exhibit osteoconductive properties but lack immediate hemostatic efficacy. This shortfall underscores the need for advanced biomaterials capable of fulfilling dual criteria: (1) limiting postoperative drainage to <20 mL/h during the critical 8–24-h window, and (2) supporting biomechanically stable callus formation within 4–6 weeks (Huzum et al., 2021). Recent innovations, including smart drainage systems with 92% sensitivity for early complication detection (Gruslova et al., 2022) and minimally invasive sternotomy techniques that reduce drainage volume by 34% (Wu et al., 2020), represent valuable adjuncts. Nevertheless, for patients with frail sternal anatomy, a composite biomaterial that simultaneously controls bleeding and accelerates bone healing may offer a solution for improving surgical outcomes.

Ezechbone® Granule CBS-400 is a Ca/P/S-based bone graft material co-developed by National Cheng-Kung University (NCKU) and Joy Medical Devices (JMD), an ISO 13485/GMP-certified facility in Kaohsiung, Taiwan. The rabbit and osteoporotic goat spine implantation studies (Yang et al., 2020a; Yang et al., 2020b) of the material demonstrate that CBS-400 integrates seamlessly with surrounding bone tissue without triggering inflammatory or other undesired tissue reactions. Fibrous tissue is rarely observed at the implant-bone interface, and the material’s early-stage new bone formation rate is more than 10 times greater than that of the autologous bone. These promising results led to its clinical use at NCKU Hospital (NCKUH) in various surgical procedures, including sternal closure, all with encouraging outcomes.

Noticeable therapeutic outcomes in sternotomy procedures at NCKUH were first observed in a patient (case 1) with a severely fragmented and unstable sternum. Not only did the sternal fragments demonstrate rapid bone healing and recovery of thoracic stability, but intraoperative observations also revealed significantly reduced blood oozing from the osteoporotic marrow void. This hemostatic outcome, combined with its excellent bone-regeneration capability readily observed in various dental and orthopedic applications, strongly suggest that CBS-400 may carry dual therapeutic functions, i.e., enhancing bone regeneration and exerting a local hemostatic effect. Motivated by these promising findings, CBS-400 was subsequently applied in two additional high-risk cases (case 2 and case 3) to further investigate its hemostatic efficacy and, at the same time, to obtain preliminary data on dosage effects on clinical outcomes on clinical outcomes. During these procedures, CBS-400 consistently exhibited strong blood absorption and clot-promoting properties when applied to marrow voids, underscoring its potential to serve as both a bone-filling and a hemostatic agent. This dual-functionality could largely help managing complex sternal reconstructions, especially in patients with compromised bone quality or infection risk. Recognizing the critical importance of developing a material with this dual functionality particularly for sternotomy application, the current study was designed to systematically evaluate CBS-400’s hemostatic performance and mechanisms in more fundamental depth. *In vitro* and *in vivo* animal studies are conducted to help interpret the clinical findings, while clinical case analysis continues to be accumulated to highlight the motivation to improve surgical success rates through this innovative, multifunctional biomaterial.

## 2 Materials and methods

### 2.1 Materials used in the study

Ezechbone® Granule CBS-400 is a fully synthetic, inorganic, resorbable Ca/P/S-based bone graft material free of animal or human source components. CBS-400 has been approved for clinical use for orthopedic and dental applications by Taiwan Food and Drug Administration (TFDA) (Approval Numbers 003889 and 003890), demonstrating its effectiveness in promoting bone regeneration. Extensive physical, chemical, and animal implantation data have been documented (Yang et al., 2020a; Yang et al., 2020b). Being substantially free from post-op adhesion and abnormal bone formation or synostosis, CBS-400 is further approved by TFDA (Approval Number 007722) for exclusive use to treat the complex intra- and peri-articular fractures near joints (Yang et al., 2022).

One commercially available Ca-based product used for comparison in this study is MBCP® (Macroscopic Biphasic Calcium Phosphate). Composed of 40% β-tricalcium phosphate (β-TCP) and 60% hydroxyapatite (HA), MBCP® is a synthetic bone substitute produced by Biomatlante® (France) and distributed by Ouqiang International Co., Ltd. This material is claimed to have a porosity design mimicking the natural microstructure of human bone, thereby enhancing its biological performance. Another commercial Ca-based product used for comparison is Q-Oss®+, also a synthetic biphasic calcium phosphate material produced by Osstem® Implant (Czechia). Composed of 80% β-TCP and 20% HA, this material is marketed for its excellent blood permeability, which promotes the migration of bone regeneration cells and supports new bone formation. Like MBCP®, the widely used Q-Oss®+ is claimed to have a microstructure similar to that of human bone, enhancing its ability to integrate with natural tissue.

### 2.2 Animal study

The animal study includes (a) an *in vitro* clotting test on rat’s blood and (b) an *in vivo* pathophysiological investigation on rat’s implantation study. The experiment has been reviewed by the Institutional Animal Care and Use Committee (IACUC, AAALAC certified) of National Cheng Kung University with Approval Number 112070.

#### 2.2.1 *In vitro* blood clotting test on rat’s blood

Two 10-week-old Sprague-Dawley rats weighting between 350 and 450 g were selected for each experiment in this study. This cardiac puncture procedure was based on the method proposed by Beeton et al. and followed the current Clinical and Laboratory Standards Institute (CLSI) guidelines, wherein a 20 mL syringe with a 22G needle was used for blood draw (Beeton et al., 2007; Lippi et al., 2017). The rats were deeply anesthetized, ensuring they showed no spontaneous movement, slow breathing, and no response to stimuli. After locating the heart, the needle was inserted at a 45-degree angle to draw 14 mL for each rat. The blood was injected into four sodium citrate tubes (VACUETTE® TUBE 3 mL Coagulation sodium citrate 3.2%, blue cap-black ring, Greiner BIO-ONE, Kremsmünster, Austria) to the full mark within 1 minute. The regular whole blood was converted to citrated whole blood by mixing gently swirling inverse to mix 5 to 6 times to maintain a non-coagulated state. The rats were then immediately euthanized.

Before whole blood collection and preparation, 28 Eppendorf tubes (tube size 1.5 mL) were prepared and divided into seven test groups. A pre-weighed 10 mg of each test material, including MBCP®, Q-Oss®+ and CBS-400, was placed in a tube. For the sham group, no material was placed in the test tubes. 100 μL of citrate-acidified blood was then added to each tube (28 tubes in total). The coagulation time of each test material and sham at 1, 2.5, 5, 9, 12, 15 and 30 min (a total of seven time points) was appointed. When the corresponding coagulation time point was completed, 1 mL distilled water was added to the tube to terminate the coagulation reaction. After standing still in the tube for 5 min, the supernatant other than the blood clot in the tube was completely aspirated and transferred to a 15 mL centrifuge tube. 3 mL distilled water was then added to the centrifuge tube to completely lyse non-entrapped red blood cells (RBCs). 200 μL of each solution was added to a 96-well plate in triplicate for Elisa reader to read the absorbance of each test sample. Six repetitions (six 96-well plates) were performed for each test group (Sham, MBCP®, Q-Oss®+ and CBS-400). In addition, 100 μL rat whole blood was added to 3 mL of distilled water, therein 200 μL solution was extracted and used as a blank group.

The optical density for absorbance at 540 nm wavelength was measured using a Microplate Reader (Multiskan FC, Thermo Fisher Scientific, Taiwan). Each sample was tested in three replicates. The blood clotting efficiency test was assessed following the reported method (Li L et al., 2020; Li J et al., 2017). Due to the fact that coagulation ability is generally inversely proportional to the absorbance value, to assess the blood coagulation ability, one focus has been assessing the effectiveness of hemostatic materials in promoting thrombus formation using BCI as a key performance parameter. The BCI was determined by calculating the ratio of the absorbance of the experimental group (measured at 540 nm, At) to that of the blank control group (measured at 540 nm, Ab) using the formula: Blood clotting index (%) = (At/Ab) × 100%. The lower the BCI, the higher the hemostatic potential of the material (Dai et al., 2022; Lan et al., 2015).

#### 2.2.2 Pathohistological examination and SEM characterization of fibrin fiber formation in rat’s tibial implantation

##### 2.2.2.1 Rat model

Since the bleeding of bone originates from trabeculae, which is the same condition in cut surface of both tibia bone and sternum, the use of tibial implantation model was used for the study to mimic the hemostasis of the study material in the sternum. Since the early-stage hemostatic efficacy is the most critical stage, in this preliminary study, the blood clot formation at the early stage (30 min) is a major focus of the study. The experiment utilized twelve 10-week-old Sprague-Dawley (SD) rats weighing 350–450 g as test animals. The aforementioned three different bone substitute materials, Ezechbone® Granule CBS-400, MBCP® and Q-Oss®+, were implanted into the proximal tibial metaphysis of rats’ hind legs. In addition to the experimental groups, rats with drilled bone defects without implantation of any material were used as a sham control group. Animal numbers and implantation time were assigned as follows: Sham group 30 min (n = 2); MBCP® group 30 min (n = 3); Q-Oss®+ group 30 min (n = 3); CBS-400 group 30 min (n = 4). The surgical procedure was based on the techniques of Kido et al. (2015). Each experimental rat was anesthetized *via* intraperitoneal (IP) injection with a mixture of Zoletil 50 and Xylazine (25 mg/kg Zoletil and 10 mg/kg Xylazine). Subcutaneous (SC) injection of nalbuphine (4 mg/kg) was administered for preoperative analgesia.

Following anesthesia the fur on the rat’s left and right hind limbs was shaved, skin incision and subcutaneous tissue dissection with hemostasis were conducted, a specific dental drill was used to drill a hole 11 mm below the joint with a diameter of 2 mm and a depth of 2 mm. This width is generally the width of the critical bone defect, while this depth is about the position of the red bone marrow where the medullary blood comes out of the cancellous bone. As a non-survival procedure, the rats were maintained under deep anesthesia for 45 min post-implantation and were subsequently euthanized *via* carbon dioxide (CO_2_) inhalation. CO_2_ was delivered at a flow rate of 30%–70% of the chamber volume per minute for at least 5 min until the absence of respiration and heartbeat was confirmed (Kim and Kim, 2013).

##### 2.2.2.2 SEM characterization of fibrin fibers in clots

The SD rats were sacrificed using carbon dioxide euthanasia according to the planned implantation time points. After removing both hind limb tibiae, the specimens were fixed in formalin (neutral buffered formalin solution, 10%, Sigma-Aldrich) for 24 h. The decalcification process followed the method described by Okamoto et al., 2018; Okamoto et al., 2018) The fixed tissues were placed in Morse’s solution for 7 days. After decalcification, the tissues were rinsed with running water for 15 min to remove any residual decalcifying solution. After decalcification in Morse’s solution, tissue dehydration, embedding, and sectioning were performed by the Human Biobank at the National Cheng Kung University Hospital. Subsequently, consecutive sections of 5 µm thickness were obtained using a Leica RM2235 microtome and were mounted onto resin blocks for scanning electron microscopy (SEM) sample preparation, or onto glass slides for hematoxylin and eosin (H&E) staining.

The 5 µm tissue sections were mounted onto slides and placed in a 60 °C oven for 60 min to dry. After cooling, the sections were deparaffinized using xylene, with deparaffinization steps of 5 min, 3 min, and 1 min, respectively. The areas surrounding the drilled holes were then covered with carbon adhesive, and the specimens were gold-coated using a sputter coater (Quorum Q150T ES). Gold coating was applied at a fixed current of 10 mA for 40 s. Micrographs were taken using a scanning electron microscope (SEM, JEOL JSM-6510, Japan) operated at 5 kV and 70 mA at a working distance of 10 mm. A magnification around 5,000× was found most appropriate for morphological characterization of the present fibrin fibers. An ImageJ software (version 1.53) was used to assist the analysis.

##### 2.2.2.3 Pathohistological characterization for assessing early-stage inflammation

Before staining, the slides were placed in a 60 °C oven for 60 min to enhance the adhesion between paraffin sections and glass slides, preventing detaching of the specimens during staining. H&E staining was performed following the procedures and parameters established by the Department of Surgical Pathology at the National Cheng Kung University Hospital. After staining, the slides were air-dried for 2 h prior to mounting using a synthetic resin-based mounting medium and coverslips. After that, the slides were left at room temperature for 24 h to allow the mounting medium to solidify.

A Leica DM 2500P microscope, equipped with a Leica DFC295 camera, was used for the present microscopic study. Using a transmitted white light source, the stained slides were examined at a series of magnifications from ×40 through ×400. For H&E-stained histological analysis, ×400 was mostly used to assess the inflammatory reactions at 30 min post-implantation. For more precise and meaningful assessment of the implant-derived inflammatory responses, the white WBC counts were determined by excluding the implanted material, bone debris, and blank areas. Due to their sharp contrast in the stained samples, the numbers of WBCs in ×400 fields (per high-power field, phf) were easily identified and manually counted.

##### 2.2.2.4 Statistical condition

All quantitative data are presented as individual data points, with box-and-whisker plots indicating the median, interquartile range, and minimum–maximum values. Mean values with standard deviations (SD) are additionally provided to summarize central tendency and data dispersion.

Blood clot index (BCI) values measured at multiple time points (1, 2.5, 5, 9, 12, 15, and 30 min) were analyzed using two-way analysis of variance (ANOVA) with treatment group (sham, MBCP, Q-Oss, and CBS-400) and time as fixed factors to assess the main effects of group and time as well as their interaction. When significant main effects were detected, one-way ANOVA was performed at individual time points, followed by Tukey’s honestly significant difference (HSD) test for multiple comparisons between each bone graft material and the sham control.

Quantitative parameters assessed at a single time point (30 min post-implantation)—including SEM-determined fibrin fiber density, fibrin fiber diameter, fibrin fiber area coverage, and histology-determined white blood cell (WBC) counts from H&E-stained sections—were analyzed using one-way ANOVA, followed by Tukey’s HSD *post hoc* test for pairwise comparisons among treatment groups.

The normality of data distribution was evaluated using the Shapiro–Wilk test, and homogeneity of variances was assessed using Levene’s test. No substantial violations of ANOVA assumptions were observed. All statistical tests were two-tailed, and a P value of less than 0.05 was considered statistically significant.

All statistical analyses were performed using JMP software (SAS Institute Inc., Cary, NC, United States).

### 2.3 Clinical case presentation

A total of nine patients is included for the study. The studies of the first group, including three patients operated by a cardiac surgeon and the second group, including six patients operated by another cardiac surgeon, have been approved by the institutional review board at National Cheng Kung University Hospital with approval numbers of A-ER-109-031 and B-ER-114-090, respectively.

#### 2.3.1 Three challenging cases

Chronic obstructive pulmonary disease, diabetes mellitus (DM), end-stage renal disease (especially under chronic hemodialysis), advanced age, immunosuppression, osteoporosis and emergent or prolonged cardiopulmonary bypass (CPB) use are recognized as strong risk factors of post-sternotomy malunion and mediastinitis (Meszaros et al., 2016). Patients having more than two of these risk factors are characterized with frail sternums. To highlight the hemostatic efficacy of CBS-400 in sternotomy, three frail sternums (cases 1, 2 and 3) were chosen. The surgical procedures of these three cases, all male, involved coronary artery bypass grafting (CABG), mitral valve repair, and aortic valve replacement (AVR) surgeries. The records of the three challenging cases were made with subjective observations rather than objective measurements.

To obtain a general idea about the dosage effect on hemostasis, three different doses of CBS-400, i.e., 0.5 g, 1 g and 2 g, were used in case 1, case 2 and case 3, respectively, along with a case without implanting CBS-400 as a control case. A standard wire cerclage fixation technique was used for the fixing of the sternum, except case 1 wherein the dehiscent sternum was re-fixed by Robicsek method due to the patient’s extremely vulnerable sternum and uneven anterior chest wall excursion during inspiration.

**FIGURE 1:**
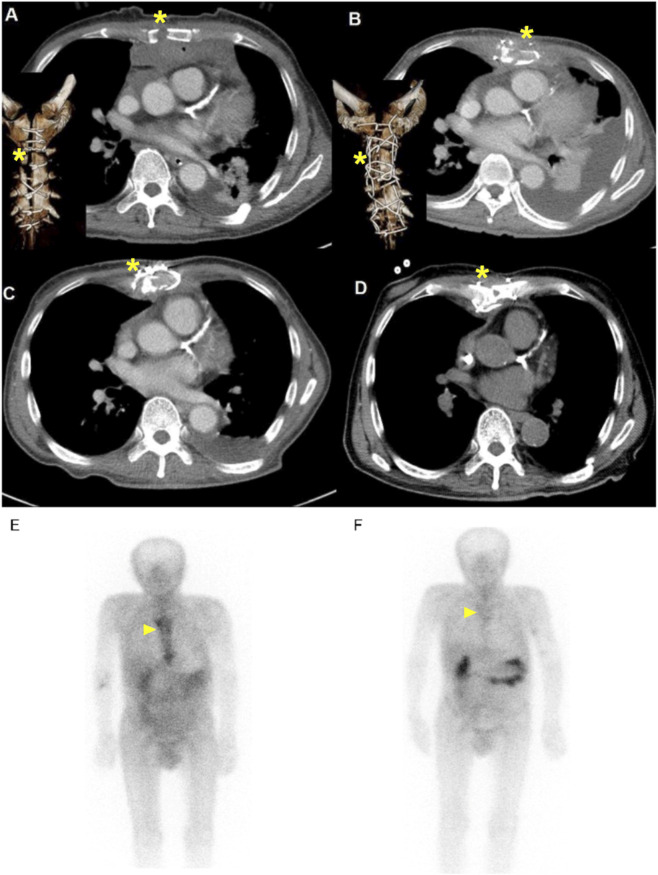
Serial images of chest computed tomography and Ga^67^ inflammation scans of case 1 using 0.5 g Ezechbone® Granule CBS-400. **(A)** 14th post-operative day after 1st operation (emergency CABG). Dehiscent sternum with wire cut-through cleft at middle sternal body (yellow asterisk). **(B)** 2 months after the 2nd operation with Robicsek fixation method. **(C)** 4 months after 2nd operation with new bone ingrowth in wire cut-through cleft. **(D)** 10 months after 2nd operation with complete bone union of sternotomy and wire cut-through cleft. **(E)** Sternal osteomyelitis indicated by increased radioactivity at the midline anterior chest area (yellow arrowhead) before 2nd operation. **(F)** Complete resolution of sternal osteomyelitis indicated by disappearance of radioactivity at the midline anterior chest area 3 months after 2nd operation.

Bone healing and rapid self-ventilation were observed after CBS-400 was applied during sternum closure in case 1, a case of severely fragmented sternum with unstable thoracis cage. During peri-operative course, much reduced blood oozing from these sternal fragments with osteoporotic bone marrow was also observed. These preliminary observations indicated that the all-synthetic, inorganic Ca/P/S-based CBS-400 had not only promoted bone healing (as will be demonstrated in Figure 1), but also reduced blood oozing of osteoporotic marrow void. Encouraged by this initial result, two subsequent complicated cases with at least two high risk factors, case 2 and case 3, were performed with use of CBS-400.

#### 2.3.2 Six consecutive cases

After the initial success, six more patients (five males and one female) were recruited consecutively from December 2024 to March 2025 for further assessment of CBS-400 hemostatic effect. These six cases include three cases as experimental group implanted with CBS-400 and three cases as control group without implant. The surgical procedures involved CABG, mitral valve repair, and AVR surgeries. The clinical study was approved by the institutional review board at National Cheng Kung University Hospital (B-ER-114-090). The success was measured based on the level of hemoglobin, chest tube drainage, and the amount of blood transfusion mainly on the post operation day 1 (Table 1).

**TABLE 1:** Peri-operation patient characteristics and clinical outcome.

Variables	Control (n=3)	Experiment (n=3)
Age	67.6 (52.5, 67.9)	57.7 (30.2, 77.5)
Gender (M:F)	3:0	2:1
Surgical procedure		
CABG	3	1
AVR	0	2^a^
Mitral repair	0	1
CKD, stage	​	​
1–2	2	2
3–4	0	1
5	1	0
DM	2	1
Heart failure, NYHA functional class	2.5 (2.0, 3.0)	3.0 (2.0, 3.0)
Hypertension	2	1
LVEF (%)	36.0 (34.2, 45.0)	65.0 (37.0, 66.3)
ESRD	1	0
Day 1 before operation	​	​
Hb	12.9 (10.4, 13.2)	13.5 (8.7, 14.5)
Plt (10^3^/µL)	154.0 (78.0, 244.0)	235.0 (221.0, 292.0)
WBC (10^3^/µL)	7.8 (7.5, 8.4)	6.8 (4.6, 6.8)
POD 1	​	​
Hb	8.9 (8.8, 9.4)	10.5 (9.3, 11.0)
Plt (10^3^/µL)	103.0 (48.0, 174.0)	178.0 (134.0, 195.0)
WBC (10^3^/µL)	12.8 (9.3, 14.7)	12.5 (7.2, 14.0)
POD2	​	​
Hb	8.6 (8.5, 9.1)	10.5 (9.0, 10.8)
Plt (10^3^/µL)	91.0 (37.0, 164.0)	158.0 (131.0, 199.0)
WBC (10^3^/µL)	14.3 (10.2, 15.3)	14.1 (11.3, 18.2)
Chest tube drainage amount	​	​
POD 1	570.0 (130.0, 1,000.0)	120.0 (60.0, 210.0)
POD 2	210.0 (70.0, 380.0)	230.0 (50.0, 470.0)
POD 3	110.0 (100.0, 150.0)	187.5 (75.0, 300.0)
Chest tube drainage duration (days)	5.0 (4.0, 6.0)	3.0 (3.0, 11.0)
Re-exploration for bleeding patient-time	1	0
Post-OP 24 h transfusion	​	​
PRBC (U)	2.0 (2.0, 6.0)	1.0 (0, 2.0)
Fresh-frozen plasma (U)	2.0 (2.0; 6.0)	4.0 (1.0, 8.0)
Leucocyte-reduced platelets (U)	1.0 (0, 1.0)	0 (0,0)
Cryoprecipitate (U)	12.0 (0, 24.0)	0 (0,0)
ICU stay (days)	5.0 (2.0, 15.0)	2.0 (1.0, 3.0)
Hospital stay after surgery (days)	12.0 (12.0, 95.1)	12.0 (7.0, 16.0)
Hospital survival	3	3

Data are presented as median (minimum, maximum). No formal statistical comparisons were performed between groups due to small sample size.

M, male; F, female; CABG, coronary artery bypass grafting surgery; AVR, aortic valve replacement surgery; CKD, chronic kidney diseases; NYHA, New York Heart Association; LVEF, left ventricle ejection fraction; ESRD, end-stage renal disease; POD, post-operation day; Hb, hemoglobin, Plt, platelet counts; WBC, white blood cell counts; ICU, intensive care unit; PRBC, packed red blood cells; leukocyte-reduced; U, unit.

^a^ Concomitant aortic valve replacement and mitral valve repair in one patient.

## 3 Results

### 3.1 *In vitro* clotting test on rat’s blood

**FIGURE 2:**
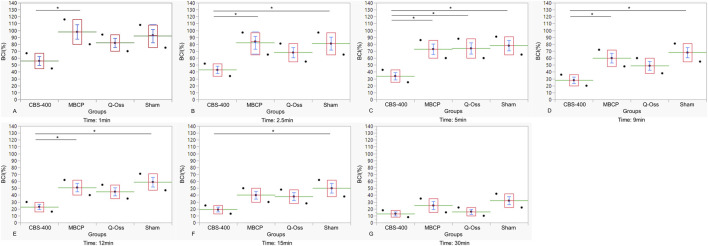
Time-dependent blood clot formation assessed by BCI values in sham and different bone graft groups at 10 mg graft/100 μL whole blood. **(A)** 1 min; **(B)** 2.5 min; **(C)** 5 min; **(D)** 9 min; **(E)** 12 min; **(F)** 15 min; **(G)** 30 min. Box-and-whisker plots (red) indicate the median, interquartile range, and minimum–maximum values. Green horizontal lines denote the mean values, and blue horizontal lines represent the standard deviations (SD). Statistical significance between groups was determined by one-way ANOVA followed by Tukey’s multiple-comparison test. P < 0.05 is indicated by one asterisk (*), and P < 0.01 by two asterisks (**).

Comparative analysis of blood clot index (BCI) values revealed distinct hemostatic behaviors among the tested groups (Figure 2). Overall, BCI values decreased over time across all groups, reflecting progressive clot formation.

Two-way ANOVA demonstrated significant main effects of treatment group (F_(3,56)_ = 37.21, P = 2.3 × 10^−13^) and time (F_(6,56)_ = 37.64, P = 6.4 × 10^−18^), whereas the group × time interaction was not statistically significant (P = 0.76).

At individual time points, one-way ANOVA identified significant between-group differences at 1, 2.5, 5, 9, 12, and 15 min (all P < 0.05), but not at 30 min (P = 0.072). Post hoc multiple-comparison analysis using Tukey’s test showed that CBS-400 consistently exhibited significantly lower BCI values than the sham control at 2.5, 5, 9, 12, and 15 min, indicating a superior and sustained pro-coagulant effect during the early to intermediate phases of hemostasis.

In contrast, no statistically significant differences were detected between the sham, MBCP, and Q-Oss groups at any time point, although the Q-Oss group tended to show slightly lower mean BCI values compared with sham and MBCP. These findings suggest that, among the evaluated bone graft materials, CBS-400 demonstrates the most pronounced hemostatic potential, whereas MBCP and Q-Oss exhibit comparable coagulation profiles to the sham control.

### 3.2 Pathohistological examination and SEM characterization of fibrin fiber formation in rat’s tibial implantation

#### 3.2.1 SEM characterization of fibrin fibers in clots

**FIGURE 3:**
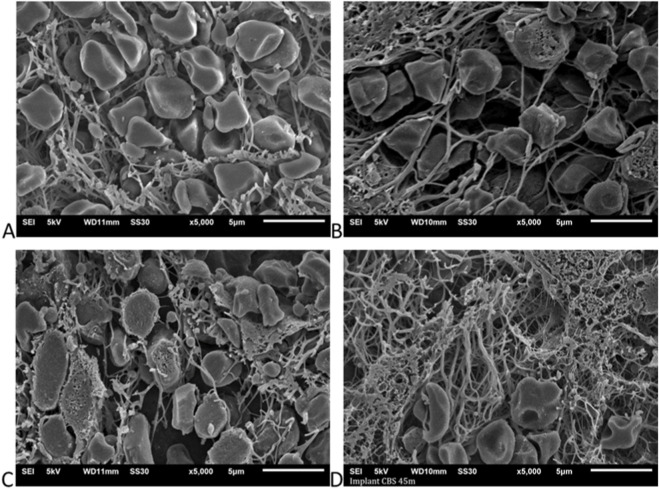
Representative fibrin fiber morphologies of sham and different bone graft groups at 30 min post-implantation under SEM. **(A)** Sham; **(B)** MBCP®; **(C)** Q-Oss®+; **(D)** CBS-400.

**FIGURE 4:**
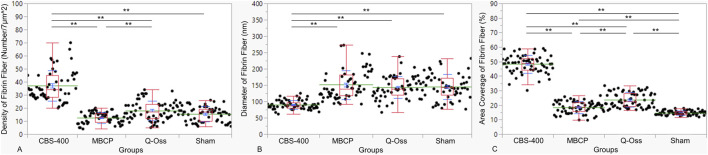
SEM-determined fibrin fiber density **(A)**, diameter **(B)** and area coverage **(C)** in all groups at 30 min post-implantation. Box-and-whisker plots (red) indicate the median, interquartile range, and minimum–maximum values. Green horizontal lines denote the mean values, and blue horizontal lines represent the standard deviations (SD). Statistical significance between groups was determined by one-way ANOVA followed by Tukey’s multiple-comparison test. P < 0.05 is indicated by one asterisk (*), and P < 0.01 by two asterisks (**).

Representative fibrin fiber morphologies of sham and different bone graft groups at 30 min post-implantation under SEM are demonstrated in Figure 3. While the fibrin fibers in all groups generally appear to be loosely packed and irregularly distributed, the fibrin fiber morphology in CBS-400 group is distinguished from other groups by its apparently higher density and coverage of the fibrin fiber network. Quantitative characterization of fibrin fibers with a comparison among all groups is given in Figure 4. As clearly indicated in the figure, CBS-400 group demonstrates significantly higher fibrin fiber density and area coverage, accompanied with a smaller fibrin fiber diameter, than sham and two other graft material groups, demonstrating a more hemostatic fibrin fiber architecture. The observed CBS-400-induced denser, thinner and broader clot networks strongly suggest its superior hemostatic and clot-stabilizing potential at the crucial early stage of surgery.

#### 3.2.2 Pathohistological analysis for early-stage inflammation evaluation

**FIGURE 5:**
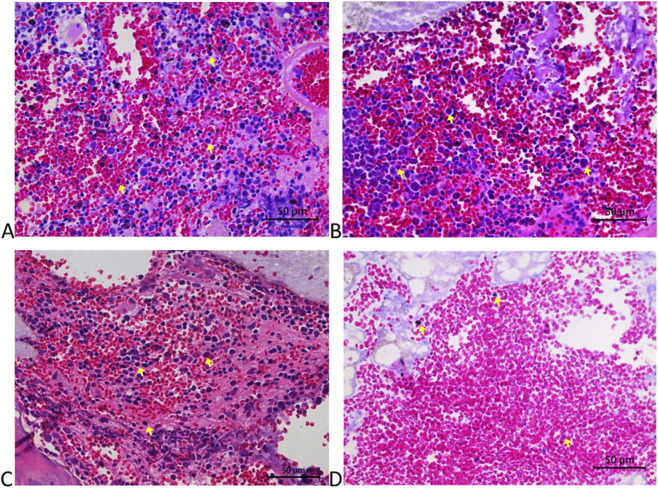
H&E-stained histology demonstrating WBCs (blue-colored cells indicated by yellow arrowheads) in Sham **(A)**, MBCP® **(B)**, Q-Oss®+ **(C)**, and CBS-400 **(D)** groups.

**FIGURE 6:**
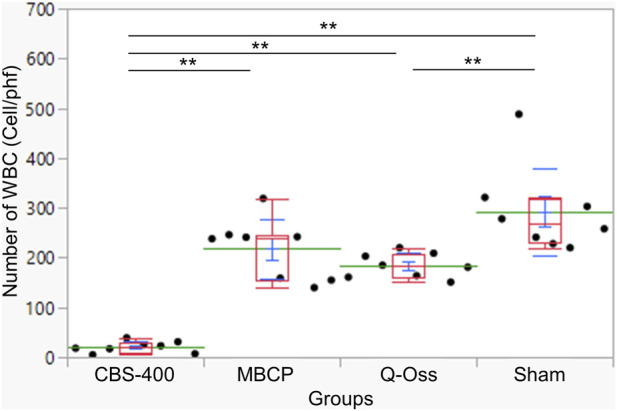
H&E-stained histology-determined WBC numbers in all groups at 30 min post-implantation. Box-and-whisker plots (red) indicate the median, interquartile range, and minimum–maximum values. Green horizontal lines denote the mean values, and blue horizontal lines represent the standard deviations (SD). Statistical significance between groups was determined by one-way ANOVA followed by Tukey’s multiple-comparison test. P < 0.05 is indicated by one asterisk (*), and P < 0.01 by two asterisks (**).

Typical H&E-stained pathohistological images of all groups are presented in Figure 5. Among all groups, Sham exhibits the largest white blood cell (WBC) number, followed by MBCP® and Q-Oss®+. Sham control fields exhibit a dense infiltration of WBCs. MBCP® and Q-Oss®+ groups show moderate cellular accumulation, indicating a relatively lower degree of acute inflammatory activity, compared to Sham. In contrast, CBS-400 demonstrates largely reduced WBC infiltration, with only scattered inflammatory cells observed across multiple fields. The same result is expectedly demonstrated in the quantitative counts of WBCs in all groups (Figure 6), therein the sham group again displays the highest mean WBC infiltration (292 ± 87 cells/phf) due to the lack of biomaterial intervention. MBCP® (218 ± 61 cells/phf) and Q-Oss®+ (184 ± 25 cells/phf) demonstrate reduced but still considerable leukocyte recruitment. By contrast, CBS-400 exhibits the lowest inflammatory cell presence, averaging only 21 ± 11 cells/phf, underscoring its ability to minimize inflammatory cell recruitment within the first 30 min of implantation.

### 3.3 Clinical results

The initial pilot three median sternotomy cases using CBS-400 are presented to assess the hemostatic effect of the investigated Ca/P/S-based bone substitute material. For a fair comparison in dosage effect on hemostatic efficacy, only complicated cases (with at least two high risk factors) are compared in depth. Control case was a 64 years/o male with coronary artery disease (CAD) post Percutaneous transluminal intervention (PCI) and poor left ventricular ejection fraction (38%). Owing to severe aortic stenosis (bicuspid valve) with dilated proximal aorta (maximal diameter 4.8 cm), he received aortic root replacement with composite vascular graft containing bovine valve and CABG x 2. The first 2-h and 4-h postoperative pericardial drainage amounts reached 180 mL and 300 mL, respectively.

CBS-400 case 1 was a 75-year-old male with critical CAD (left main and 3-vessel, LM + 3-VD) who suffered from acute myocardial infarction (AMI) with cardiogenic shock. PCI failed to resuscitate his condition. His co-morbidities included end-stage renal disease (ESRD) under hemodialysis and DM. Emergent CABG was conducted under intra-aortic balloon pumping (IABP) and extracorporeal membrane oxygenation (ECMO) support. On postoperative day (POD)-14 of the first operation, poor sternum healing was observed. The patient’s sternum appeared very vulnerable and uneven anterior chest wall excursion during inspiration was noted. Massive blood gushed out when the skin staples were removed. Emergent chest computed tomography (CT) revealed unhealed sternum with multiple fragmentations caused by cutting-through of the fixation steel wire. A 7 mm-wide gap at sternal manubrium and massive mediastinal hematoma were present (Figures 1A–D). Sternal osteomyelitis was also indicated by the radioactive gallium (Ga)^67^ inflammation scan (Figure 1E). Mediastinal tissue culture disclosed infection of methicillin-resistant *Staphylococcus aureus*, a kind of bacteria relatively hard to deal with. After emergent re-exploration and debridement of infected mediastinal tissues, the dehiscent sternum was re-fixed by Robicsek method combined with 0.5 g Ezechbone® Granule CBS-400 sprinkled on the edges of sternotomy halves and fragmented clefts. The total blood drainage on 1st, 2nd, 3rd and 4th day was 170, 170, 100 and 70 mL, respectively. The last tube was removed on 5th day. The patient recovered after 2nd op and was discharged from hospital on 14th day after operation. The sternal wound was stable without respiratory distress. Complete resolution of sternal osteomyelitis was indicated by the disappearance of radioactivity at the midline anterior chest area 3 months after 2nd op (Figure 1F).

CBS-400 case 2 was a 64-year-old male with DM nephropathy. He suffered from relapsing congestive heart failure (New York Heart Association, NYHA, function class, Fc, III) and was confirmed to have CAD (LM + 3-VD). CABG x three was therefore conducted. Severe osteoporosis of his sternum was found during operation. 1 g CBS-400 was used and the drainage was carefully measured by the hour. The blood drainage of the two chest tubes on 1st and 2nd h was 40 mL each. On the 3rd hour, trivial drainage was observed. The total blood drainage on POD-1 was only 160 mL.

CBS-400 case 3 was a 74-year-old diabetic male who had a huge aortic aneurysm with the maximal diameter of 7.1 cm extending from ascending aorta, aortic arch, and proximal descending aorta. 80% stenosis of right coronary artery (RCA) orifice was also noted. The patient received total ascending aorta and arch replacement combined with frozen elephant trunk graft stenting at the proximal descending aorta. RCA bypass was conducted with great saphenous vein graft. Innominate, left carotid and left subclavian arteries were revascularized with prosthetic grafts. The overall CPB time, circulatory arrest time and aortic clamping time were 250 min, 30 min and 40 min, respectively. For this complicated surgery, 2 g CBS-400 was used during sternal closure accompanied with a standard wire cerclage fixation technique. A video file is provided to demonstrate the sternum closure procedure using CBS-400. The blood drainage from two pericardial tubes at postoperative 1st and 2nd h was further reduced to 20 mL each. At the 3rd hour, again, the bleeding substantially stopped, leading to a total POD-1 blood drainage being only 80 mL. All three patients received CBS-400 recovered smoothly after operation.

The video file (Supplementary Material-Video File 1S) demonstrates the hemostatic effect of CBS-400 in the sternum closure procedure. When the sternum closure procedure was conducted in the absence of CBS-400, a sucking tube was usually used to keep sucking out excessive blood to maintain a clear surgical field. With CBS-400, however, gauze was all needed from time to time to wipe out the slowly seeped-out blood.

In the subsequent six consecutive cases, the follow-up and comparative evaluation included three patients in the control group and three in the experimental group (Table 1). The median age was higher in the control group (67.6 years) compared to the experimental group (57.7 years). The control group consisted entirely of male patients, whereas the experimental group included two males and one female. Surgical procedures differed between the groups. All control patients underwent CABG, while the experimental group consisted of two patients who underwent aortic valve replacement (AVR) and one who received mitral valve repair. Preoperative comorbidities were described in Table 1. Chronic kidney disease (CKD) stages 1–2 were present in two patients per group, while one control patient had CKD stage 5. Diabetes mellitus was observed in two control patients and one experimental patient. The New York Heart Association (NYHA) functional class was 3.0 (median) in the experimental group, and 2.5 (medium) in the control group. The median left ventricular ejection fraction (LVEF) was 36.0% in the control group, and 65.0% in the experimental group.

There was one episode of re-exploration for bleeding in the control group in one patient, while none in the experimental group, Table 1. During the post-operation day 1, the control group received a median of 2 unit packed red blood cells (PRBC), 2 units of fresh frozen plasma (FFP), 1 unit of platelet, and 12 units of cryoprecipitate. The experimental group received only 1 unit of PRBC and 4 units of FFP without the need for platelet and cryoprecipitate transfusion.

Regarding hematological parameters, on the day before surgery, the medium hemoglobin (Hb) levels were 12.9 g/dL and 13.5 g/dL forthe control and the experimental group, respectively. Postoperatively, Hb levels dropped in both groups, with values of 8.9 g/dL and 8.6 g/dL respectively in the control group on POD 1 and 2. The medium levels were 10.5 g/dL and 10.5 g/dL in the experimental group Platelet counts also decreased postoperatively on POD 1 and POD 2.

In postoperative drainage and outcomes, chest tube drainage volumes on POD 1 were 570 mL (medium) and 120 mL for the control and the experimental group, respectively. On POD 2, the median drainage volumes were 210 mL and 230 mL for control and experimental group, respectively. On POD 3, the median drainage amount in experimental group was 187.5 mL, and the control group was 110 mL. This is explained by one case of post-pericardiotomy syndrome in the experimental group. The duration of chest tube drainage was 5.0 days (medium) in the control group, and 3.0 days in the experimental group.

Finally, the length of the intensive care unit (ICU) stay was 2.0 days (medium) in the experimental group, and 5.0 days in the control group. The prolonged ICU stay in the control group was primarily associated with one patient’s need for ECMO. Postoperative hospital stays were 12 days (medium) in both groups. All patients survived to hospital discharge.

## 4 Discussion

Hemostasis, a process preventing blood loss from a damaged vessel and promoting healing, is known to critically depend on the formation of an effective fibrin fiber network. This network originates from fibrinogen, a protein synthesized in the liver, which, upon thrombin activation, undergoes cleavage to form fibrin monomers. These monomers subsequently self-assemble and polymerize into fibrin fibers, serving as a vital scaffold for further cellular activities and tissue repair (Mihalko et al., 2021; Weisel and Litvinov, 2013; Levy et al., 2012; Mihalko et al., 2021; Weisel and Litvinov, 2013; Levy et al., 2012. The diameter of these fibrin fibers is a critical factor influencing the mechanical properties of the resultant clot as well as its biological functionality (Li et al., 2016; Gray et al., 2011). This phenomenon is essential during the initial hemostatic response as fibrin clots serve to occlude the breach in the vascular system, thereby averting exsanguination. Observations have shown that both density and structure of the fibrin fibers-whether thick and loosely packed or thin and tightly packed can modulate the effectiveness of hemostasis (Weisel and Litvinov, 2013; Litvinov et al., 2019; Gu and Lentz, 2018). Moreover, the diameter of fibrin fibers can be influenced by various factors. For example, increased thrombin levels typically promote the rapid polymerization of fibrin monomers into thinner fibers, while lower thrombin activity can lead to the formation of thicker fibers due to the lower polymerization rate (Zubairova et al., 2015; Piechocka et al., 2016).

In the present study, the *in vitro* coagulation assays confirm that CBS-400 accelerates thrombus formation significantly faster than Sham, MBCP® and Q-Oss®+, as reflected by its consistently lower BCI across all time points. This may at least partly explain why Sham group elicits more acute inflammation than other groups, i.e., by physically entrapping more WBC cells in the clots. SEM evaluation corroborated these findings, revealing that CBS-400-promoted denser, thinner, and more extensive fibrin networks are key determinants of clot strength and stability. According to Wang et al., fibrins with a higher density and smaller diameter demonstrate a better clotting ability than those with a lower density and larger diameter (Wang X et al., 2016).

Inflammation has been recognized as another crucial phase for the initial-stage bone healing process, setting the groundwork for subsequent regenerative events. Immediately following a fracture, the rupture of blood vessels leads to hematoma formation, providing a provisional matrix and serving as a site for cellular infiltration (Zhou et al., 2021) This Inflammation phase is characterized by a complex cascade of cellular and molecular interactions, including the recruitment of such immune cells as neutrophils, macrophages and lymphocytes, each plays an essential role in tissue repair and regeneration (Loi et al., 2016; Wendler et al., 2019; Maruyama et al., 2020; Qiu et al., 2024). During this stage, a concurrent increase in WBC count is commonly observed, reflecting the systemic inflammatory response to tissue injury. Elevated WBC levels, primarily attributable to neutrophil and macrophage infiltration, are crucial for combating potential infections, clearing necrotic debris, and initiating the healing cascade (Kawai et al., 2021; Bastian et al., 2011). The inflammatory response further promotes the recruitment of mesenchymal stem cells (MSCs) and progenitor cells, facilitating the transition towards the repair phase (Qiu et al., 2024; Maruyama et al., 2020) In addition, growth factors such as basic fibroblast growth factor (bFGF) are reported to involve in promoting vascular ingrowth, which is essential for soft callus formation, the next phase of healing (Zhou et al., 2021). While such mediators as interleukin-1β (IL-1β) and tumor necrosis factor-alpha (TNF-α) are indispensable for early repair (Loi et al., 2016; Schmidt-Bleek et al., 2014; Kon et al., 2001), they also contribute to pain by sensitizing peripheral nociceptors, leading to impaired mobility and potential secondary complications, if inadequately managed (Sarifah et al., 2021; Huang et al., 2023).

On the other hand, the resolution of inflammation, a necessary step to prevent chronic inflammatory conditions that may impair healing, is also considered crucial (Maruyama et al., 2020; Wendler et al., 2019; Xu et al., 2023). Persistent high levels of inflammation can lead to altered healing dynamics, resulting in delayed or impaired bone repair. The severity of inflammatory response and the overall state of bone healing are often reflected in the pattern and composition of WBCs in circulation. For example, a systemic inflammation, signified by the increased WBC counts and associated inflammatory markers, can directly influence bone healing outcomes. The inter-relationship between WBCs and other entities involved in inflammatory response highlights the significance of timely resolution of inflammation to facilitate progression to the repair phase of bone healing (Loi et al., 2016; Maruyama et al., 2020). It is also reported that the successful resolution of this initial inflammatory phase is vital for optimal transition to the repair and remodeling phases, with evidences suggesting that dysregulation can lead to impaired healing outcomes (Maruyama et al., 2020; Wendler et al., 2019; Li et al., 2023).

From these referenced discussions, it can be seen that the inflammatory phase of bone healing is a complex interplay of immune responses for clearing the initial damage and setting the stage for repair. It is essential for both pro-inflammatory and anti-inflammatory mediators to work in harmony, while any perturbations in the balance of inflammatory responses can lead to delayed healing (Kemmler et al., 2015; Rapp et al., 2016).

The results of the present study indicate that CBS-400 elicits the mildest acute inflammatory response among all tested materials, as evidenced by its significantly reduced WBC infiltration both qualitatively in histological images and quantitatively in numerical counts. Compared to two other Ca-based bone substitute materials tested in the study, MBCP® and Q-Oss®+, which produce moderate leukocyte responses, CBS-400 demonstrates a clear advantage in biocompatibility at the early implantation stage. The present animal and clinical studies suggest that, at least for the present Ca/P/S-based material, the benefits from its reduced inflammatory response apparently outweigh the aforementioned pro-inflammatory effects.

From a material point of view, the hemostatic and fast postoperative bone healing effects of CBS-400 are related to a careful design in its chemistry, crystal structure, overall morphology and porosity population, size and distribution, as well as a built-in fluid dynamic consideration. All such biocompatibility tests as cytotoxicity, intracutaneous reactivity, skin sensitization and subchronic tests have demonstrated good biocompatibility features of CBS-400 (Yang et al., 2020b). This study also indicates that CBS has a greater resorption rate than other implant materials, such as beta-TCP (Gauthier et al., 2001), biphasic calcium phosphate (60% HA/40% TCP) (Le Guehennec et al., 2005) and deproteinized bovine bone (Chakar et al., 2014) under similar implantation conditions (NZW rabbit femur condyle).

The synthetic, inorganic CBS-400 seems to demonstrate osteoinductive features yet without drawbacks of common organic/osteoinductive products. CBS-400 seems to have largely bypassed the inflammation route by quickly and directly facilitating new bone formation throughout the entire implantation site, even in the deep interior of the implant at the very early stage. The built-in high porosity level (70–80 vol%) of CBS-400 particles provides a larger blood-contacting surface area when implanted into a bone defect. Large amounts of ions, such as calcium which is well-known able to assist bleeding management, can be quickly released from this large surface into blood. This factor alone may at least partly explain the hemostatic performance of CBS-400. As mentioned earlier, osteoporosis is also one key risk factor of sternal dehiscence. CBS-400 has also demonstrated a high potential to treat osteoporotic fractures. In an osteoporotic goat spine implantation study (Yang et al., 2020a), it was found that the trabecular bone architecture of the implanted goat had substantially recovered to the normal level in about 6 months after implantation without signs of osteoporosis-related delay in the bone maturing process.

From the clinical point of view, conventional sternotomy has been a widely applied surgical approach in the life-threatening cardiovascular procedures, notably for valvular replacement and acute aortic syndrome. However, these techniques are often associated with complications, including significant blood loss and prolonged ICU stays. For instance, one study reports that patients undergoing conventional sternotomy exhibit an average blood loss of 360 mL in the first 24 h, leading to heightened requirements for blood transfusions, which can introduce risks such as transfusion reactions and increased infection rates (Berretta et al., 2022). The relationship between blood loss and ICU duration is evident. Excessive bleeding may necessitate additional interventions, extend ICU stays and complicate recovery paths (Park et al., 2012). Furthermore, complications from increased blood loss, such as hemodynamic instability, can place further strain on recovery resources in ICU, potentially influencing both procedural outcomes and hospital stay. No doubt, effectively managing blood loss is paramount in improving surgical outcomes and promoting earlier ICU discharges (Zhang et al., 2021). In this small, uncontrolled pilot series, the CBS-400 group showed lower median drainage volumes, though group differences in surgical procedures, cardiac function, and comorbidities preclude any causal interpretation. In a retrospective cohort midterm follow-up study from two centers on sternal healing after median sternotomy in low-risk patients, (Wang B et al., 2019), observed that, even all patients were given non-absorbable bone wax for sternal hemostasis, the mean POD-1 drainage volume was 450.0 mL for plates-fixed cases (N = 25) and 421.3 mL for wire-fixed cases (N = 19). The two different fixing methods did not show much differences. Compared to these data, the POD-1 drainage volumes are much lower in the present CBS-400-implanted cases even with higher risks. The same study of Wang et al. also indicates that patient’s age, but not closure device (plate or wire), is a risk factor for sternal non-healing. Neither is the presently observed CBS-400-involved bone healing efficacy in case 1 considered as a result of its different wiring method used.

Additional possible mechanism underlying the hemostatic effect of CBS-400 may involve CBS-400-induced charged surface favorable for platelet adhesion and activation (Yang et al., 2020a; Yang et al., 2020b; Yang et al., 2022), while its porous architecture enhances local blood absorption and fibrin entrapment. The present results show that CBS-400 may provide a clinical advantage in high-risk cardiothoracic and orthopedic procedures.

Despite the various encouraging results of the study, several limitations should be acknowledged. The animal and clinical sample sizes remain modest to show the causal efficacy, and larger randomized controlled studies are warranted to establish definitive efficacy and safety profiles. The long-term effects of CBS-400 on infection resistance and remodeling dynamics require further investigation. Furthermore, the observed CBS-400-derived dual efficacy in hemostasis and bone regeneration suggests that these two critical factors could be closely related to each other, especially at the crucial early stage of healing, which invites further basic research on this subject.

## 5 Conclusion

CBS-400, a fully synthetic Ca/P/S-based bone graft substitute, has demonstrated hemostatic efficacy and osteoregenerative potential in both preclinical and clinical settings. It significantly accelerated clot formation, promoted effective fibrin fiber network development, minimized acute inflammatory responses, and improved perioperative outcomes in frail sternotomy patients. By addressing both bleeding control and bone healing, CBS-400 represents a novel multifunctional solution with the potential to improve surgical outcomes in patients at risk of postoperative bleeding, or bone healing complications. Future larger-scale clinical studies are needed to confirm these findings and to establish standardized guidelines for its optimal use.

## Data Availability

The raw data supporting the conclusions of this article will be made available by the authors, without undue reservation.

## Supplementary material

SUPPLEMENTARY VIDEO 1 The video file demonstrates the hemostatic effect of CBS-400 in the sternum closure procedure. When the sternum closure procedure was conducted in the absence of CBS-400, a sucking tube was usually used to keep sucking out excessive blood to maintain a clear surgical field. With CBS-400, however, gauze was all needed from time to time to wipe out the slowly seeped-out blood.
